# Autonomic dysfunction in epilepsy mouse models with implications for SUDEP research

**DOI:** 10.3389/fneur.2022.1040648

**Published:** 2023-01-06

**Authors:** Jennifer Bauer, Orrin Devinsky, Markus Rothermel, Henner Koch

**Affiliations:** ^1^Department of Epileptology and Neurology, RWTH Aachen University, Aachen, Germany; ^2^Institute for Physiology and Cell Biology, University of Veterinary Medicine Hannover, Foundation, Hannover, Germany; ^3^Departments of Neurology, Neurosurgery and Psychiatry, NYU Langone School of Medicine, New York, NY, United States

**Keywords:** epilepsy, SUDEP, brainstem, mouse models, cardiorespiratory activity

## Abstract

Epilepsy has a high prevalence and can severely impair quality of life and increase the risk of premature death. Sudden unexpected death in epilepsy (SUDEP) is the leading cause of death in drug-resistant epilepsy and most often results from respiratory and cardiac impairments due to brainstem dysfunction. Epileptic activity can spread widely, influencing neuronal activity in regions outside the epileptic network. The brainstem controls cardiorespiratory activity and arousal and reciprocally connects to cortical, diencephalic, and spinal cord areas. Epileptic activity can propagate trans-synaptically or *via* spreading depression (SD) to alter brainstem functions and cause cardiorespiratory dysfunction. The mechanisms by which seizures propagate to or otherwise impair brainstem function and trigger the cascading effects that cause SUDEP are poorly understood. We review insights from mouse models combined with new techniques to understand the pathophysiology of epilepsy and SUDEP. These techniques include *in vivo, ex vivo*, invasive and non-invasive methods in anesthetized and awake mice. Optogenetics combined with electrophysiological and optical manipulation and recording methods offer unique opportunities to study neuronal mechanisms under normal conditions, during and after non-fatal seizures, and in SUDEP. These combined approaches can advance our understanding of brainstem pathophysiology associated with seizures and SUDEP and may suggest strategies to prevent SUDEP.

## Epilepsy and SUDEP

Epilepsy affects ~0.75% of all people (1), or ~50 million people worldwide, with an incidence of 4–10/1000 people/year. Epileptic seizures result from abnormal hypersynchronous neuronal activity (2, 3). Most seizures arise from both hemispheres simultaneously (generalized) or from restricted regions in one or both hemispheres but can propagate widely (focal) (4, 5). Anti-seizure medicines (ASMs) prevent seizures for ~67% of patients, but many well-controlled patients experience cognitive and behavioral comorbid disorders and ASMs side effects. One-third of patients have drug-resistant epilepsy and often take multiple and high doses of ASMs with greater comorbidities, adverse effects, impairments of quality of life, and higher mortality (6–8).

Sudden unexpected death in epilepsy (SUDEP) is a witnessed or unwitnessed, non-drowning, and non-traumatic death in a person with epilepsy which often but not always follows a convulsive seizure. SUDEP excludes status epilepticus and cases where post-mortem examination or toxicology reveals another cause of death (9). SUDEP is the leading cause of death in drug-resistant epilepsy (DRE), with an incidence rate of 1–5 cases per 1000 patients per year (10, 11). SUDEP is the second leading neurological cause of lost years of life after stroke (12). Case-control studies reveal the following risk factors: generalized tonic-clonic seizures (GTCS) (any in the last year and further increased risk with ≥ 3/year), lack of adequate medication, nocturnal seizures, and lack of nocturnal supervision (13, 14). Many SUDEP cases are undetected or misclassified, suggesting the incidence is higher than reported (13, 15).

The few SUDEPs recorded on video with electroencephalographic and electrocardiographic data are biased toward more severe focal epilepsy cases admitted for presurgical evaluation with rapid ASMs reduction (16). By contrast, SUDEP affects the full spectrum of people with epilepsy (17), and results from epilepsy monitoring units cannot be generalized. The underlying mechanisms of SUDEP remain poorly defined. Most occur during sleep and follow convulsive seizures, with reduced brain activity and respiratory impairments commonly observed, although cardiac dysfunction can contribute (18–20). Postictal disruption of brainstem regulation of arousal, respiratory and cardiovascular functions is considered the common final pathway of death in SUDEP (9, 20–23). In animal models, arousal and cardiorespiratory dysfunctions can result from fast direct synaptic circuit mechanisms (24–27) and slower phenomenons like spreading depressions (SD) (28–31). How cortical seizure activity impairs brainstem functions postictally is a critical research issue. Understanding this pathophysiology will inform preventative and therapeutic strategies. We review potential mechanisms of seizure propagation and spread that might contribute to SUDEP, examine models used to study the mechanisms, and highlight advances in investigating complex network interaction *in vivo* in mouse models.

## Propagation of epileptic activity – the problem of a highly connected brain

This section reviews the following questions: how does epileptic activity spread to the brainstem? Is this a rare event, or common but usually compensated for (and if so, how)?

Rodent, primate, and human brains orchestrate multiple areas to optimally assess internal and external conditions and determine behavioral outputs. This requires high connectivity, precise coordination, and balance between interacting cortical-subcortical networks. During cortical seizures, affected areas are directly impacted by aberrant excitation and inhibition. In addition, areas beyond the epileptic network can be severely disturbed by ictal spread to resonating areas. The brainstem receives projections from cortical and subcortical brain areas (32–34). During and after seizures, these connections can alter brainstem activity and potentially impair arousal and cardiorespiratory functions and contribute to SUDEP (32, 34–36). Understanding why some cortical seizures propagate to other cortical and subcortical areas and how this disrupts brainstem activity is a major challenge in SUDEP research. The brain regions involved in epileptic circuits - cerebral cortex (37), hippocampus (38), amygdala (39, 40), and thalamus (41) - are directly and indirectly connected to the brainstem and exert powerful influences over it. The brainstem and more rostral cerebral regions share strong reciprocal connections, complicating our understanding. We review new techniques to study network interaction involved in SUDEP in epileptic mouse models.

## General concepts of the spreading of pathological activity

Epileptic seizures can be provoked by disrupting neuronal E/I balance by altering intrinsic properties, or by altering synaptic transmission and network stability causing hypersynchronous activity (42). The mechanisms underlying seizure propagation and termination are less well characterized. Focal seizures influence other brain areas *via* rapid axonal connections or spreading depression (SD), a slow propagating depolarization wave that inactivates neurons (25, 31, 35, 36). This slow ictal wavefront propagation corresponds to the gradual evolution of seizure symptoms, as in the Jacksonian sensory symptom march (43). The ictal wavefront may evoke a feedback loop to the seizure focus which triggers the clinical symptoms. Failure of feedforward inhibition supports epileptiform activity and seizure spread *via* this slow route in addition to classic synaptic pathways (44, 45). While SD contributes to symptoms of migraine and epilepsy, the mechanisms may be conserved or divergent (29, 46). The propagation rate of SD in migraine and epilepsy are similar, but their onset, duration, impacted brain regions and EEG changes can differ (47–50). Different SDs might exert distinct influences on brainstem function and SUDEP risk (29, 51). Debate persists whether this risk is primarily an ictal or post-ictal phenomenon. While the ictal seizure spreading into the brainstem might cause direct autonomic dysfunctions (36, 52, 53), the disturbance in the post-ictal period might substantially outlast the seizures. The post-ictal EEG suppression is viewed as a potential contributor but only a weak SUDEP predictor (54–56).

Mouse models of familial hemiplegic migraine with mutations in *Cacna1a* (57, 58), *Atp1a2* (59) and *Scn1a* (60, 61) show increased mortality. In mice with *Cacn1a* variants, brainstem SD elicited by seizures can be fatal (31). Brainstem SD may directly impair cardiorespiratory function (21, 30, 31, 35, 40). In focal seizures, SD with seizure propagation may be restricted to cortical regions in most instances. SDs were directly triggered by high neuronal activity of focally induced seizures and prevented by applying tetrodotoxin (TTX; a potent sodium channel inhibitor) (62). The authors postulate that SD is an innate mammalian mechanism to prevent seizure propagation and generalization, and to induce seizure termination (62). However, if SDs reach brainstem autonomic centers, severe cardiorespiratory dysfunction may follow (31, 63).

## Brain areas linked to autonomic control

Human studies used electrical stimulation or the time of seizure invasion to investigate cortical structures that alter breathing. These brain areas include the amygdala (32, 36, 64), the hippocampus head and body, anterior parahippocampal gyrus, and antero-mesial fusiform gyrus (65, 66). A pediatric study found apneas and seizure spread to the amygdala were strongly correlated (67), an adult study failed to replicate this (64). Electrical stimulation to the insula and left cingulate gyrus decreased cardiac output and induced cardiac asystole in epilepsy patients without effects on breathing (68, 69). However, electrical activation cannot precisely target specific neurons and circuits. Further, cortical and subcortical electrode coverage is limited. So the invasion of ictal activity to a region (e.g., amygdala) may be accompanied by spread to areas that were not sampled (e.g., hypothalamus, anterior cingulate, and orbitofrontal cortices). Also, correlating seizure invasion to apneas might reveal only some parts of the network involved in autonomic dysfunction. In animals and humans, physiological changes in subcortical areas (e.g., locus coeruleus) alter breathing (27, 70).

In addition to seizure invasion of cortical areas, altered connectivity between cortical areas and respiratory brainstem centers may be important (71). Functional magnetic resonance imaging (fMRI)-studies on epilepsy patients show reductions in resting-state functional connectivity and tissue loss in cortical, subcortical, and brainstem structures associated with impaired autonomic control and increased SUDEP risk (71–73). However, monitoring of patients who later died from SUDEP did not reveal a direct associated location or lateralization of the epileptogenic zone with their higher risk of death (16). Intracranial EEG recordings and stimulation studies implicate the amygdala, hippocampus, insular cortex, and seizure spread to the contralateral temporal lobe to correlate with ictal cardiorespiratory dysfunctions (32, 36, 53, 65, 67, 74). Thus, identifying the detailed and complex connectivity and the altered brain activity in regions controlling cardiorespiratory activity is crucial for SUDEP risk estimation.

## Control of autonomic functions in the brainstem and SUDEP

**Figure 1:**
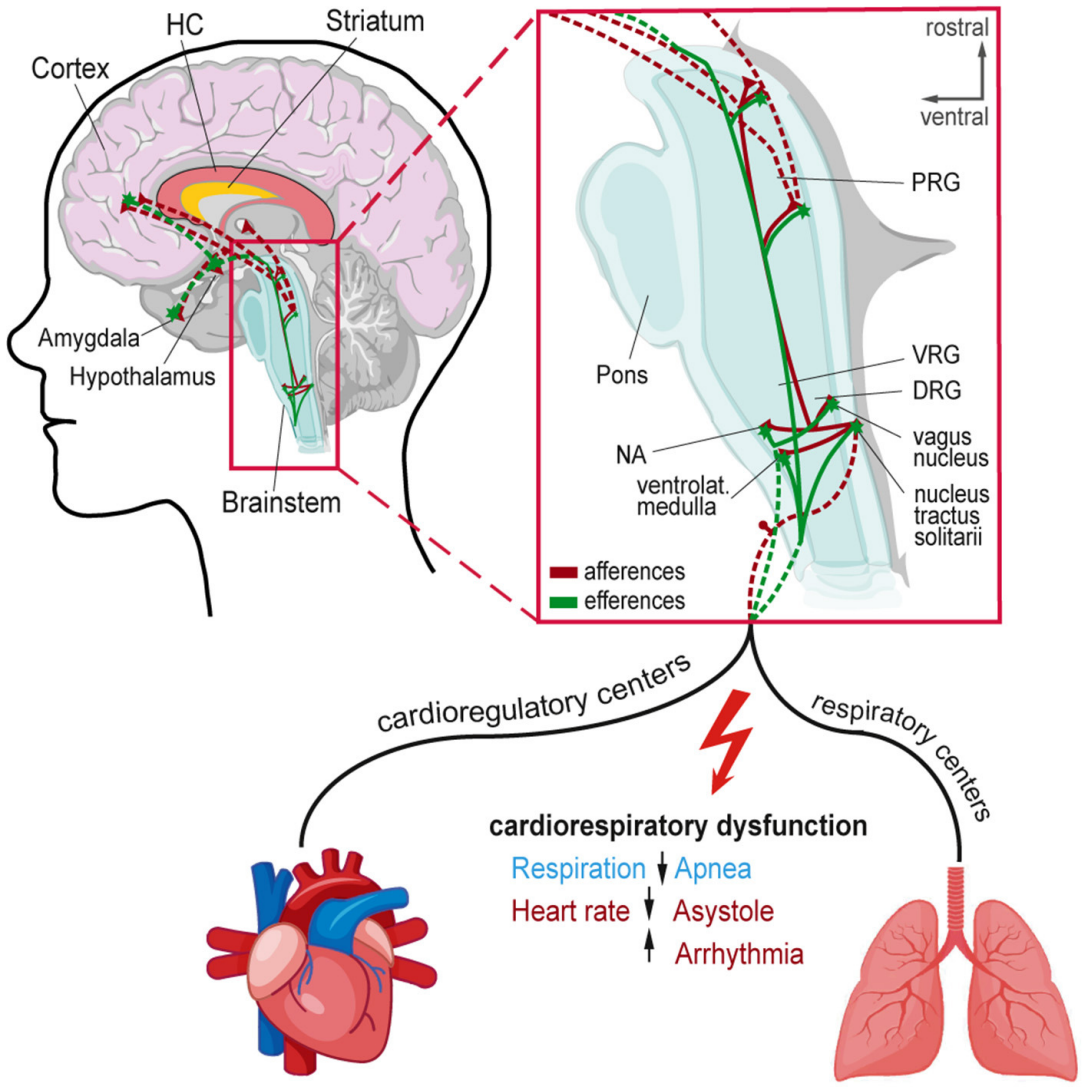
Afferent and efferent connections to central autonomic networks and proposed mechanisms involved in seizure-induced cardiorespiratory dysfunction leading to SUDEP. Cortical and subcortical regions involved in epileptic activity and cardiorespiratory modulation in the brainstem. Magnification shows connections and brainstem nuclei of cardioregulatory and respiratory centers that can be affected by seizure activity leading to cardiorespiratory dysfunction. DRG, Dorsal respiratory group; HC, Hippocampus; NA, Nucleus ambiguous; PRG, Pontine respiratory group; VRG, Ventral respiratory group.

**Figure 2:**
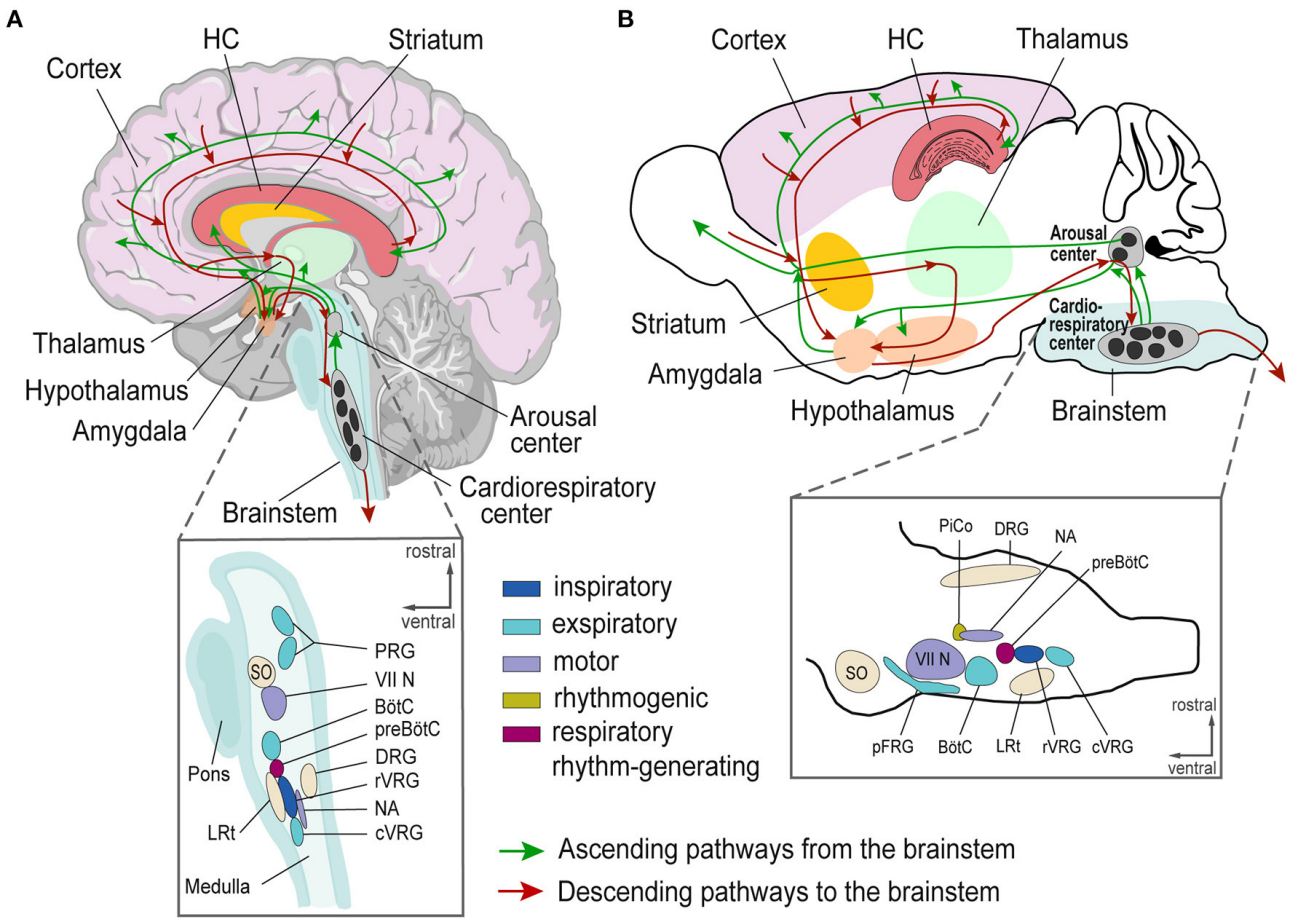
Schematic of brain regions involved in epileptic activity and cardiorespiratory regulation in humans **(A)** and mice **(B). (A, B)** Top, Human and mouse brain with depicted Cortex (pink), Hippocampus (red), Striatum (yellow), Thalamus (green), Hypothalamus (orange), and brainstem (blue) with ascending (green lines) and descending (red lines) projections. Bottom, Magnification of brainstem nuclei. VII N, facial nucleus; BötC, Bötzinger Complex; cVRG, caudal ventral respiratory group; DRG, Dorsal respiratory group; HC, Hippocampus; LRt, lateral reticular nucleus; NA, Nucleus ambiguus; pFRG, para-facial respiratory group; PRG, Pontine respiratory group; PiCo, Post-inspiratory Complex; preBötC, preBötzinger Complex; rVRG, rostral ventral respiratory group; SO, superior olive; VRG, Ventral respiratory group.

Here, we review evidence of brainstem alterations (e.g., genetic or physiological/structural resulting from chronic epilepsy) associated with SUDEP risk. We discuss crucial brainstem areas generating and modulating autonomic rhythms, such as breathing, and discuss their potential role in SUDEP. The respiratory network flexibly adapts to environmental and metabolic changes while maintaining stability to guarantee effective gas exchange (75). This network integrates brainstem rhythm-generating nuclei with other central and peripheral neural regions (76). The brainstem respiratory network includes the parafacial respiratory group (pFRG), Bötzinger complex (BötC), pre-Bötzinger complex (pre-BötC), rostral ventral respiratory group (rVRG), and caudal VRG (cVRG). Pontine nuclei modulate respiratory activity *via* projections to medullary respiratory nuclei (34, 76, 77). The post-inspiratory complex (PiCo) provides excitatory input to generate post-inspiration patterns (78). Seizure-related effects on respiratory and cardiac brainstem centers can impair these functions and contribute to SUDEP (20, 21, 79, 80) (Figure 1). Cardiorespiratory dysfunction in SUDEP could result from the effects of higher cortical and limbic areas on brainstem function, direct brainstem alterations, or both, including descending and ascending circuitries (27, 38, 81) (Figure 2). Chronic alterations of respiratory control, such as reduced ventilatory responses to increased CO2 levels, occur in epilepsy patients (82).

Epileptic seizures can directly alter heart rhythms and heart rate variability (HRV), which reflects balanced sympathetic and parasympathetic activity (83–86). High sympathetic tone and elevated levels of several neuropeptides can follow seizures (87, 88). Other seizure-induced acute changes include asystole, brady- and tachy-arrhythmias are most common with seizure foci in paralimbic and limbic cortices (69) and may contribute to SUDEP (23, 89, 90). Reduced HRV can result from voltage-gated sodium channel gene variants (91), and low-frequency HRV power is associated with SUDEP risk (92). Temporal lobe seizures may disturb arousal and vigilance networks (93).

A critical challenge is distinguishing indirect vs. direct effects on brainstem autonomic centers. For example, PreBötC dysfunction can result from mutations in ion channels (94–96) and mitochondrial genes (97), as well as transcription factors (98). In animal SUDEP models with *Kcna1* and *Scn1a* mutations, the threshold to trigger brainstem SD is reduced (21). However, respiratory networks are state-dependent; neuromodulators influencing respiratory activity include norepinephrine, serotonin, acetylcholine, substance P, ATP, somatostatin, dopamine, endorphins, and adenosine (99). Several have been shown to be elevated during and following seizures and potentially could contribute to SUDEP (88, 100–102).

The brainstem is crucial for controlling cardiorespiratory autonomic function impairments likely contribute to sudden infant death syndrome (SIDS), the sudden and unexpected death of a seemingly healthy baby under age 1 year (103–105). There are striking similarities between SIDS, sudden unexplained death in childhood (SUDC), and SUDEP (106–109) with the exclusion of other causes, nocturnal occurrence in the prone position, and an unwitnessed death (106). Arousal can be triggered by increased CO_2_ (hypercapnia) and reduced oxygen levels (hypoxia), further preventing a build-up of end-tidal CO_2_ and restoration of normal oxygen levels (103). This arousal response is linked to breathing and is normally initiated with a sigh (augmented breath) (110–112). Sighs are generated in the PreBötC by the same rhythm-generating network crucial for eupnea and gasping (95, 113). In addition, several other areas such as the dorsal raphe nucleus, the nucleus tractus solitarius, the parabrachial nucleus, and the retrotrapezoid nucleus are involved in arousal (106). Seizures in the amygdala [bed nucleus of the stria terminalis (BNST)] can activate projections to the brainstem, disturbing structures like the parabrachial nucleus involved in arousal and respiratory function (40). The BNST is highly interconnected to cortical regions, the hippocampus, the hypothalamus, the midbrain, and other brainstem nuclei and may serve as an integrator of autonomic and neuroendocrine responses (40, 114–117). As discussed above, massive release of neuromodulators (e.g., norepinephrine, serotonin, and acetylcholine) can disturb arousal. Since hypoxia and hypercapnia trigger arousal and gasping, they are a focus of SIDS research. However, another vulnerability phase is reoxygenation after a hypoxia/hypercapnia. This phase includes post-hypoxic ventilatory depression (118, 119), which can occur after generalized tonic-clonic seizures and could be potentially prolonged in SUDEP. To dissect these mechanisms, modern experimental technology, including optogenetics and chemogenetics in animal models, as discussed below, is critical.

Future directions of SUDEP research seek to identify common molecular and cellular changes overlapping in several SUDEP animal models and potentially identify common changes in SIDS and SUDC models. While expression changes in RNA levels in brainstem areas of animals showing SD in cortical areas were detected (120), more investigations in epileptic animal models (genetic and induced) are needed to unravel molecular changes that participate in SUDEP.

## ASM and brainstem function

Another potential SUDEP mechanism is direct ASM effects on brainstem function. ASM can reduce SUDEP risk by a reduction of seizure frequency and severity, thereby preventing seizure-induced impairment of brainstem autonomic centers. Under normal oxygen concentrations, mammals are eupneic, their robust respiratory network combines diverse synaptic and intrinsic signals in the respiratory network (99). During severe hypoxia, the respiratory network generates gasping (121, 122) through reduced mechanisms of rhythm generation (99, 113). During gasping, changes include reduced inhibition (123) and a switch to sodium-dependent intrinsic neuronal bursting securing rhythm generation (124–126). These altered rhythm-generating properties of the respiratory network alter the sensitivity to sodium channel-blocking ASMs and may interrupt the gasping response during seizure-induced postictal hypoxia (127). These direct brainstem effects may contribute to increased mortality associated with lamotrigine use observed in some studies (128). Moreover, during seizures, patients can experience repeated hypoxic episodes combined with increased norepinephrine and other neurotransmitter/modulator levels. This combination can destabilize PreBötC function (129) and may parallel secondary changes induced by the hypoxic conditions during and after cortical seizures.

More investigations of the brainstem function and gene expression of the brainstem areas controlling respiratory and cardiac functions are needed in epileptic mice to get a better understanding of the mechanisms underlying SUDEP. New insights into chronic brainstem changes could stem from novel techniques of brainstem transcriptome using single-cell RNA-Seq or spatial transcriptomics (130).

## Model systems for studying epilepsy and SUDEP

Model systems help to investigate the pathological interactions between brain regions that can result in a collapse of cardiorespiratory function and SUDEP. *In vitro* and *in vivo* models can study neuronal disease network mechanisms (131–133). *In vitro* models standardize experimental conditions, but oversimplify neuronal network function or whole organism interaction, which may be critical in SUDEP. *In vivo* models comprise diverse methods and model organisms (134–138). Brain areas involved in epileptic activity and cardiorespiratory regulation are similar in mice and humans (78, 139) (Figures 2A,B). Thus, mice can appropriately model human epilepsy. Modern techniques can target specific brain areas and predefined neuronal cell populations to decipher their role in epilepsy and SUDEP (140).

**Figure 3:**
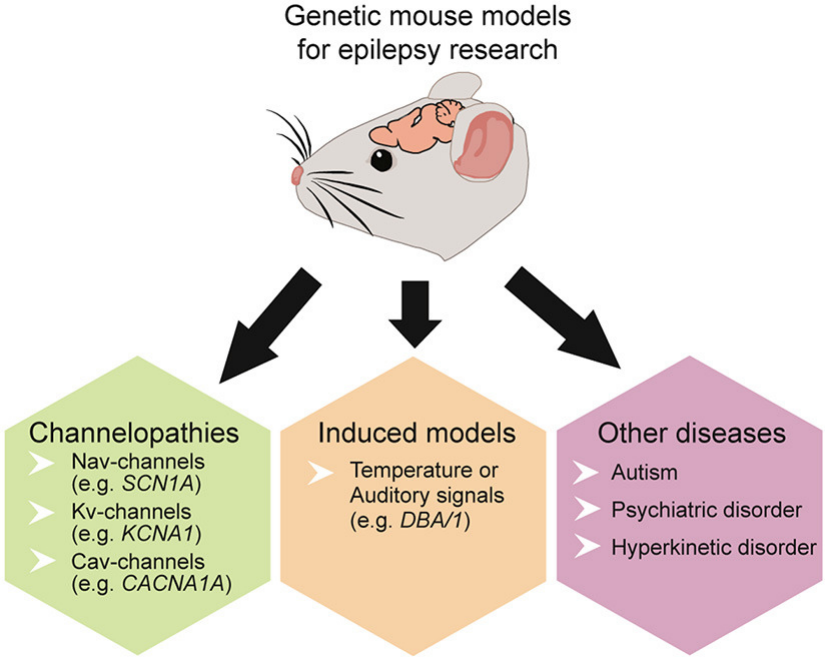
Overview of commonly used genetic mouse models to study epilepsy, SUDEP, and co-existing disorders.

Epilepsy can result from diverse pathological processes, including trauma, stroke, tumors, infections, autoimmune disorders, and >150 genetic variants (141, 142). Epilepsy is often accompanied by comorbid disorders, including autism spectrum, cognitive, psychiatric, and hyperkinetic (143–148). The developmental and epileptic encephalopathies (DEEs) include a diverse spectrum of early-life epilepsies, often resulting from genetic disorders, and associated with developmental delays partly attributable to seizures and interictal epileptiform activity (149). Across these disorders, E/I imbalances occur in the amygdala, cortex, hippocampus, and other epileptogenic regions (150, 151). Complex and heterogeneous genetic mouse models recapitulate various human pathologies, offering insights into epilepsy and SUDEP and allowing controlled experiments on mechanisms by controlling for different confounds. Epilepsy mouse models are divided into induced and genetic models. In kindling models, stimulation (electrical, chemical, or acoustic) induces seizures, whereas in genetic models, gene mutations result in spontaneous seizures (136, 152). Mouse models can mimic focal and generalized epilepsies as well as post-traumatic epilepsy (153), temporal lobe epilepsy (TLE) (152), genetic variants (80, 154), and scores of rare genetic disorders (e.g., tuberous sclerosis complex, CDKL5, Rett Syndrome, Dravet Syndrome, FOXG1 syndrome, STXBP1 syndrome and many more) (Figure 3).

Some genetically modified mouse lines model SUDEP with deadly seizure-induced cardiorespiratory abnormalities (39, 155). Many genetic models involve ion channels, including sodium voltage-gated channels (Na_v_) (154, 156–158), potassium voltage-gated channels (K_v_) (28, 159, 160), or calcium voltage-gated channels (Ca_v_) (30, 161) (Table 1). Some display spontaneous epileptic seizures (e.g., *Scn1a, Scn1b, Kcna1, Kcnq1, Cacna1a, Shank3*) (31, 143, 159, 162, 163) while others are susceptible to heat or audiogenic-induced seizures (e.g. Scn1a, Scn8a, DBA/1) (80, 164) (Figure 3).

**Table 1:** Overview of common channelopathies in mouse models of epilepsy and SUDEP.

**Gene**	**Channel**	**Expression**	**Disorder**	**Studies**
SCN1A	Na^+^ channel (α subunit of Na_v_1.1)	Central nervous system and cardiac myocytes	Genetic epilepsy with febrile seizures plus (GEFS+), Dravet Syndrome	(22, 169, 170, 173, 174, 191)
SCN1B	Na^+^ channel (β subunit of Na_v_1.1)	Central and peripheral nervous system, skeletal, and cardiac muscles.	Genetic epilepsy with febrile seizures plus (GEFS+)	(163, 181)
SCN8A	Na^+^ channel (α subunit of Na_v_1.6)	Central nervous system	Epilepsy	(148, 154, 164, 182)
KCNA1	K^+^ channel (α subunit of K_v_1.1)	Central and peripheral nervous system	Epilepsy, Episodic ataxia	(159, 160, 183, 187)
KCNH2	K^+^ channel (K_v_11.1)	Brain and heart	Long QT syndrome	(165, 189)
KCNQ1	K^+^ channel (K_v_7.1)	Heart, intestinal cells	Long QT syndrome	(162)
CACNA1A	Ca^2+^ channel (α subunit of Ca_v_2.1)	Brain	Epilepsy, Familial hemiplegic migraine, Episodic ataxia	(30, 31, 35, 161)

Commonly SUDEP mouse models carry mutations in the Na_v_ (*1.1, 1.6*) and K_v_ *(1.1, 7.1, 11.1)* genes (159, 162, 165). *Scn1a* mutations alter the Na_v_α1 subunit (Na_v_1.1) and Na_v_1.1 haploinsufficiency can cause Dravet Syndrome (DS). DS is a treatment-resistant early-onset epilepsy with 70-80% of cases due to *Scn1a* variants and high rates of SUDEP (9, 83, 166–169). Na_v_1.1 is expressed in inhibitory neurons. A loss of function decreases their excitability, increasing network excitability, altering action potential (AP) dynamics (170–173) and impairs thalamic glutamatergic and GABAergic function, disrupting thalamocortical networks and facilitating seizure generation (174, 175). Na_v_1.1 deficient mice recapitulate many aspects of human DS pathology including severe epilepsy, multiple neuropsychiatric comorbidities, and increased SUDEP risk (21, 22, 83, 173, 176–180). Other gene mutations (e.g., *Scn1b* and *Scn8a)* display similar symptoms (163, 164, 181, 182). Mice with mutations in genes encoding for K_v_ show cardiorespiratory failure including cardiac abnormalities and apnea observed in SUDEP (21, 183). K_v_1.1-α1 subunits, encoded by the *Kcna1* gene, are crucial for neuronal excitability and are broadly expressed in the cortex, hippocampus, cerebellum, and brainstem (184, 185). *Kcna1* knockout mice display early-onset generalized tonic-clonic seizures, seizure-related death, and cardiorespiratory dysfunction (159, 186–188). These mice exhibit apneas, increased respiratory variability, and precede cardiac failure as risk factors for SUDEP (183, 187). Further K_v_-channelopathies (e.g., *Kcnh2* and *Kcnq1*) are susceptible to recurrent seizures and long QT syndrome (LQTS); i.e., arrhythmias and SUDEP (162, 189, 190).

Mutations in genes encoding for Na_v_1.1, K_v_1.1, and Ca_v_2.1 are moreover linked to brainstem seizures, medullary SD, and cortical seizures propagating to the brainstem causing cardiorespiratory arrest (21, 30, 31, 35). Thereby, local brainstem SD can elicit EEG suppression, apnea, bradycardia, and asystole, mimicking the involvement of SD in epileptic activity propagation and its relevance as SUDEP models.

Thus, a number of model systems and especially mouse models, are nowadays available for epilepsy and SUDEP research. In the direct context of SUDEP, models with Na_v_ (*1.1, 1.6*) and K_v_ *(1.1, 7.1, 11.1)* mutations seem particularly promising. Of these, *Scn1a* models have been extensively studied and largely model the human SUDEP pathology and phenotypes well (152, 167, 191). Future studies need to extend to clinically and genetically characterized epilepsies to explore if common or distinct pathways of autonomic dysfunction mediate SUDEP.

## Techniques to study network interaction involved in SUDEP

To understand SUDEP mechanisms, we need models and techniques to represent and measure cortical seizure generation and propagation as well as cardiorespiratory function. *In vivo* techniques allow direct epileptic activity measurements and manipulations (192, 193) of complex circuitries and brain connections. *Ex vivo* recordings from targeted brain regions allow cellular processes to be investigated at high resolution. *Ex vivo* measurements like histological reconstructions, stainings, and spatial transcriptomics (180, 194) can reveal anatomical brain changes associated with epilepsy, which may be the cause or effect of epilepsy or an epiphenomenon of the underlying pathology.

**Figure 4:**
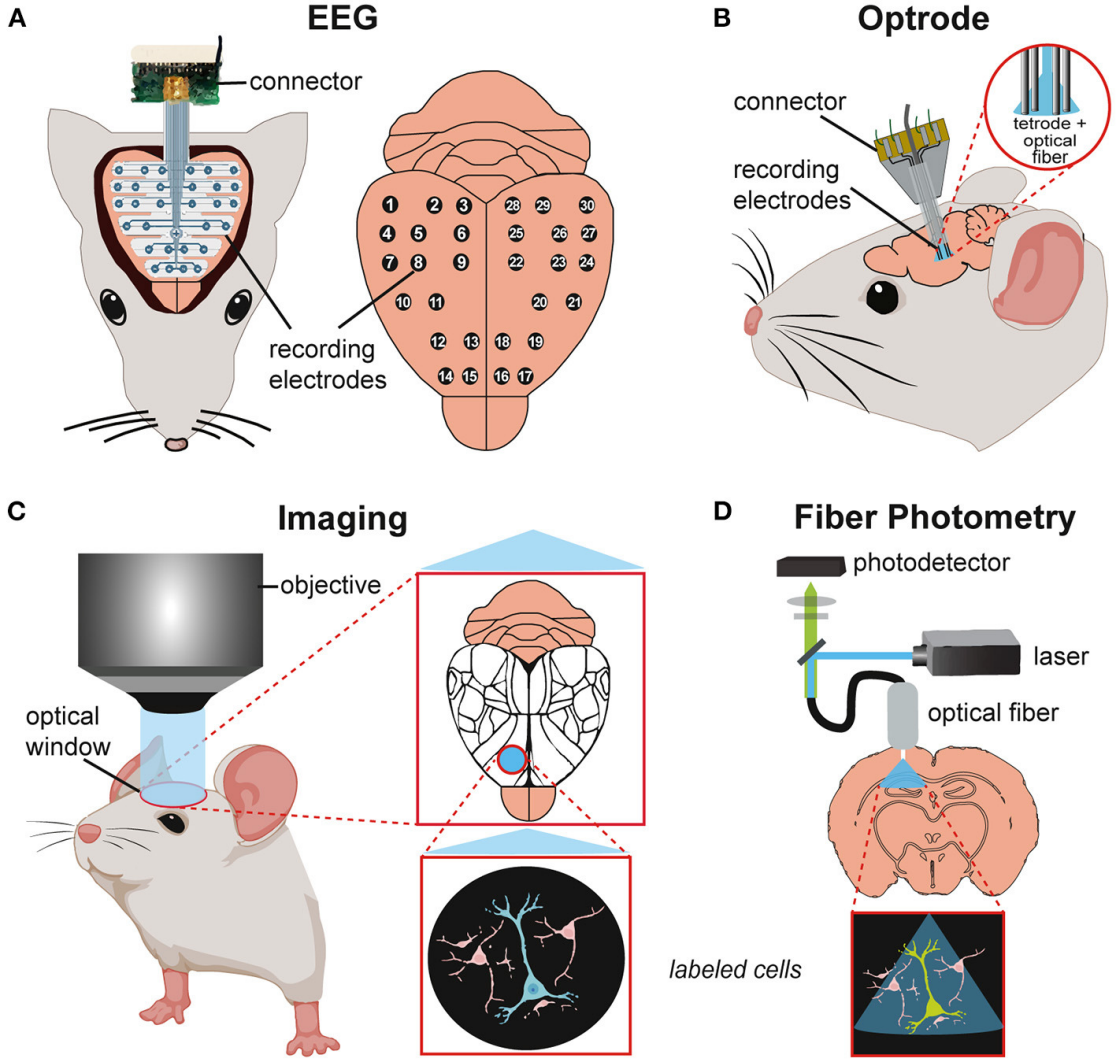
Schematics of *in vivo* techniques for studying neuronal activity in mouse models. **(A)** Electroencephalography (EEG) recording with implanted EEG probe and recording electrodes (30 channels) on the brain surface. **(B)** Implated optrode with integration of optical fiber and recording electrodes for simultaneous optical stimulation and electrophysiological recordings. **(C)** Imaging of the mouse brain through an optical window for visualizing brain areas (mapping, Allen Brain Atlas) or/and single-cell activity in the region of interest. **(D)** Fiber photometry of a target brain region with simultaneous optical stimulation (laser) and calcium imaging (photodetector) via an implanted fiber probe.

Next, we will discuss recent advancements in methods to investigate *in vivo* and *ex vivo* models, including optogenetics, electrophysiology, imaging, and other measurements (Figure 4).

Optogenetics is a technique to study specific cells and their relations to brain functions and disorders (195). Optogenetics utilizes the expression of light-sensitive proteins (opsins) in brain areas or specific cells. Depending on the opsin used, targeted neurons can be activated or inhibited (or even both) using light stimulation to precisely control neuronal activity. Optogenetics can trigger or prevention of epileptic activity (196) with millisecond temporal precision, enabling the assessment of how specific firing patterns affect brain cells and networks (197). Optogenetics can be applied invasively and non-invasively (198) and can be combined with electrophysiological recordings and imaging techniques. Chemogenetics can selectively modulate cellular pathways using restricted artificial chemogenetic receptors [e.g. DREADDs (Designer Receptors Exclusively Activated by Designer Drugs)] delivered to specific neuronal populations. Instead of light stimulation, chemogenetics systemically injected or microinfused can activate ligands that excite or inhibit targeted neurons (199). Optogenetics can be combined with chemogenetics to manipulate neuronal activity with a high temporal and spatial resolution (200). In epilepsy animal models, these combined methods can identify and manipulate specific neuron populations, brain regions, and neuronal circuitries involved in epileptic activity (201).

Seizures and interictal epileptic discharges (IEDs) can be restricted to certain brain regions and networks. Electroencephalographic (EEG) recordings can localize brain regions giving rise to seizures and examine epilepsy-related neuronal activity changes across brain regions (202). Scalp EEG records changes in electrical potentials caused by ion flow across neural membranes, mainly at the brain's surface. It can detect the origin and propagation of epileptic activity throughout different brain regions at a macro scale (203, 204) (Figure 4A). Invasive methods include intracranial EEG (iEEG) using depth or subdural EEG recordings (ECoG) to study seizure onset and spread as well as SD and seizure propagation in SUDEP models at a higher spatiotemporal resolution (22, 30). Stereotaxically inserted multi-channel electrodes can record local field potentials (LFPs) and single-cell activity (204, 205). Modulation of neuronal activity *via* electrical stimulation with these electrodes is possible but is much less precise than optogenetic manipulation. For example, SDs can be induced by electrical, and optogenetic techniques (206) whereas electrophysiological and optical recording methods can assess their propagation and effects on other structures (207, 208).

Combined optogenetics and electrophysiology *in vivo*, using optical microelectrodes are called *optrodes* (209, 210) enabling a direct readout of manipulated cell activity. Here, a single microelectrode probe with integrated optical fiber can simultaneously record and transmit light to genetically modified, opsin-expressing cells. Optrodes can study neuronal circuit dynamics in awake-behaving animals (211, 212) (Figure 4B).

Imaging allows the visualization/mapping of cortical activity with high spatial and temporal resolution (213, 214). Voltage-sensitive dyes (VSDs) or genetically encoded calcium indicators (GECIs) react to direct or indirect (Ca^2+^) changes in neuronal activity. VSD imaging incorporates dyes into the cell membrane that signal membrane-potential differences as changes in fluorescence. VDS imaging can monitor synaptic transmission and propagation of cortical activity but has a low signal-to-noise ratio and lacks cellular specificity (215, 216). GECIs allow cell-specific targeting but have a slower temporal resolution. GECIs have been used to record population activity in wide-field calcium imaging experiments. Combined with two-photon imaging, GECIS can reveal activity dynamics of hundreds of individual neurons (217). Since the activity-dependent changes in calcium-sensitive proteins can be visualized *in vivo* over months, large-scale longitudinal functional studies can assess activity before, during, and after seizures and in a single animal (Figure 4C). Imaging techniques can visualize seizures *in vivo* at high temporal and cellular resolution (172, 218).

Fiber photometry can combine imaging and optogenetics using an implanted fiber-optic cannula to deliver excitation pulses and monitor activity-dependent fluorescence changes (219). This technique is ideal for deep brain recordings and can study calcium signals in distinct epileptic brain regions in freely moving mice (220) (Figure 4D). Fiber photometry can be used simultaneously with electrophysiological recordings to combine cell-type-specific imaging with high temporal-resolution spike recordings in freely behaving mice (221).

These methods have provided new insights into the role of brain regions and cell populations in epilepsy. Optogenetics combined with optical manipulation, and electrophysiological recordings revealed the key role of inhibitory GABAergic interneuron signaling in seizure generation and ictal propagation in epileptic mice (212, 222). Other studies addressing brainstem excitatory neurons showed a direct correlation to reduced subcortical activity during seizures (223). Together, these techniques provide new research opportunities on epilepsy networks and seizure dynamics over the whole brain.

**Figure 5:**
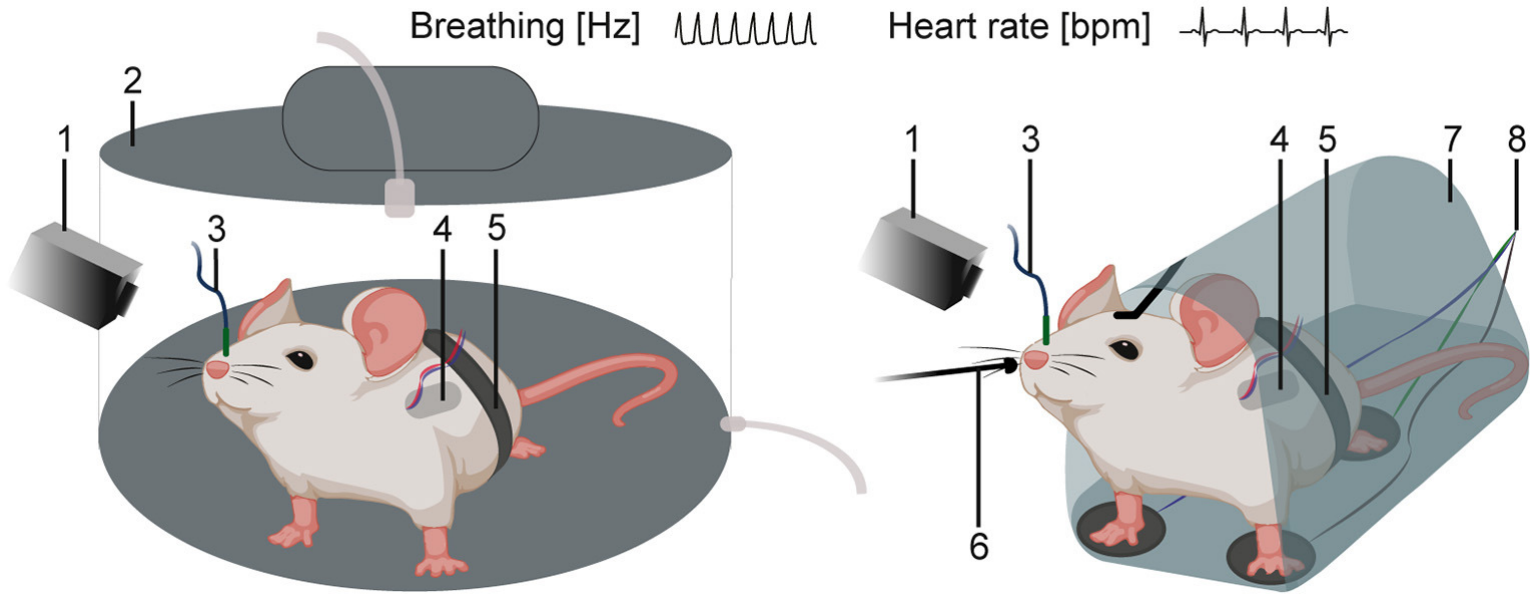
Schematics of *in vivo* techniques for the recording of cardiac and respiratory activity. Mouse in a whole-chamber plethysmograph **(left)** or in a restraining system **(right)** with additional recording methods. (1) video monitoring and infrared camera, (2) whole-chamber plethysmography, (3) intranasal cannula, (4) telemetry system, (5) movement sensor, (6) external temperature probe (thermocouple), (7) restraining system, and (8) 3-point ECG.

Investigating SUDEP and cardiorespiratory dysfunctions requires additional recording techniques for *in vivo* monitoring of autonomic functions including breathing and heart rate. Several methods are available in the mouse (155, 182, 183, 224). Cardiac activity is typically recorded *via* electrocardiography (ECG) (225, 226). Methods can monitor breathing (227) including invasive (telemetry systems and intranasal cannulas) and non-invasive methods (movement sensors, restraining systems, plethysmographs) (Figure 5). The whole-chamber plethysmography approach offers a non-invasive method in freely, non-restrained animals (95, 97, 228). This technique allows recordings of breathing under hypoxia/hypoxemia conditions (low blood oxygen levels and insufficient oxygen supply) linked to SUDEP (30, 52, 229).

Although *in vivo* methods provide insights into the network mechanisms, *ex vivo* studies offer more focused investigations of cellular changes. Histological reconstructions and stainings of brain regions can follow *in vivo* experiments to verify transgene expressions and precisely localize implanted electrodes or optical fibers (30, 180, 230). Brain slice preparations containing cortical, hippocampal, or brainstem microcircuits allow single-cell recordings or small network analysis to gain insights into pathophysiology (78, 95, 231, 232). Spatial transcriptomics can map the organization and connectivity of distinct genetically defined cell types (194, 233). In epilepsy research, this can provide a deeper exploration of disease mechanisms and pathogenic changes in the spatial organization and molecular signaling networks (234).

Thus, combining different techniques can provide a greater definition of the dysfunctions associated with epileptic activity and its interplay with autonomic functions on different levels to identify possible biomarkers for epilepsy, seizure onset, and SUDEP (202, 235).

Possible therapeutic approaches could be based on electrical or optical stimulation of specific brain areas to “rebalance” their E/I activity and maintain cardiorespiratory function during and after seizures (198, 236). Electrical stimulation in patients to map epileptic zones can inhibit or enhance respiration (237). Optogenetic neuronal activation has been shown to suppress seizure-induced respiratory arrest and exert an anticonvulsant effect in a SUDEP mouse model (238). Further, *ex vivo* methods might provide opportunities for new molecular targets and drug screening (233, 234, 239). However, these invasive approaches will require far more refinement for their potential benefits to exceed their definite risks.

## Conclusion and future perspectives

SUDEP is the leading epilepsy-related cause of death, affecting all age groups and epilepsy severities. SUDEP mechanisms are poorly understood but are critical for preventive and therapeutic strategies. Although ASMs can control seizures in most patients, they do not alter long-term prognosis or cure epilepsy (240). Further, their side effects can be severe (241, 242). 30% of the patients with ASM-resistant epilepsy suffer ongoing seizures and experience an increased SUDEP risk. Medications/treatments that prevent seizures in those that are currently uncontrolled with minimal side effects are desperately needed. Understanding SUDEP mechanisms in more detail is a desperate need.

Epilepsy mouse models with ion channel mutations mimic human epilepsies (176) and are critical in translational neuroscience research (243). They offer possibilities to investigate the link between genetic alterations and their underlying neurobiological mechanisms in much greater detail compared to humans. Translation of basic animal research to human epilepsy is exemplified by *SCN1A*-mice whose response to ASM has enabled the development of FDA-approved medications and gene therapy trials (191). Translational research with new molecular targets for anti-epileptogenic and anti-seizure research can empower novel drug discoveries and identify potential biomarkers for early diagnoses and more effective treatments (235, 243, 244).

Cardiorespiratory inhibition following epileptic seizures may be the common final mechanism of SUDEP. Cardiorespiratory dysfunctions from cortical or subcortical epileptic activity propagating to brainstem regions could cause SUDEP (21, 30, 31). SD might be directly involved in SUDEP-related seizure spread to the brainstem (29). Mouse models combining technological advances allow precise investigations of the brain networks implicated in SUDEP (235). These brain areas may provide new targets for interventions to prevent SUDEP.

In mice, invasive methods such as optical or electrical stimulations can manipulate neuronal networks (198, 245) whereas neurostimulation-based techniques can also be applied to epilepsy patients. Acute and chronic deep brain stimulation (DBS), as well as vagus nerve stimulation (VNS), are epilepsy therapies (236, 246–248). Combining neurostimulation and ASM may be more effective in controlling seizures than either alone (249).

There remains a critical need to better understand the mechanisms of epilepsy and SUDEP. Mouse models combined with precise methods are an important tools to assess these mechanisms and translate this knowledge into preventive and therapeutic strategies.

## Author contributions

JB, OD, MR, and HK reviewed the literature and wrote this review article. All authors contributed to the article and approved the submitted version.
